# Enhanced Adhesion of *Campylobacter jejuni* to Abiotic Surfaces Is Mediated by Membrane Proteins in Oxygen-Enriched Conditions

**DOI:** 10.1371/journal.pone.0046402

**Published:** 2012-09-28

**Authors:** Sheiam Sulaeman, Mathieu Hernould, Annick Schaumann, Laurent Coquet, Jean-Michel Bolla, Emmanuelle Dé, Odile Tresse

**Affiliations:** 1 INRA UMR1014 SECALIM, Nantes, France; 2 LUNAM Université, Oniris, Université de Nantes, Nantes, France; 3 Université de Rouen, Laboratoire Polymères Biopolymères Surfaces, UMR 6270 and FR 3038 CNRS, IFRMP23, Mont-Saint-Aignan, France; 4 UMR-MD1, Université de Aix-Marseille, IRBA, Facultés de Médecine et de Pharmacie, Marseille, France; Charité, Campus Benjamin Franklin, Germany

## Abstract

*Campylobacter jejuni* is responsible for the major foodborne bacterial enteritis in humans. In contradiction with its fastidious growth requirements, this microaerobic pathogen can survive in aerobic food environments, suggesting that it must employ a variety of protection mechanisms to resist oxidative stress. For the first time, *C. jejuni* 81–176 inner and outer membrane subproteomes were analyzed separately using two-dimensional protein electrophoresis (2-DE) of oxygen-acclimated cells and microaerobically grown cells. LC-MS/MS analyses successfully identified 42 and 25 spots which exhibited a significantly altered abundance in the IMP-enriched fraction and in the OMP-enriched fraction, respectively, in response to oxidative conditions. These spots corresponded to 38 membrane proteins that could be grouped into different functional classes: (i) transporters, (ii) chaperones, (iii) fatty acid metabolism, (iv) adhesion/virulence and (v) other metabolisms. Some of these proteins were up-regulated at the transcriptional level in oxygen-acclimated cells as confirmed by qRT-PCR. Downstream analyses revealed that adhesion of *C. jejuni* to inert surfaces and swarming motility were enhanced in oxygen-acclimated cells or paraquat-stressed cells, which could be explained by the higher abundance of membrane proteins involved in adhesion and biofilm formation. The virulence factor CadF, over-expressed in the outer membrane of oxygen-acclimated cells, contributes to the complex process of *C. jejuni* adhesion to inert surfaces as revealed by a reduction in the capability of *C. jejuni* 81–176 Δ*CadF* cells compared to the isogenic strain.

Taken together, these data demonstrate that oxygen-enriched conditions promote the over-expression of membrane proteins involved in both the biofilm initiation and virulence of *C. jejuni*.

## Introduction

*Campylobacter* is one of the major causative agents of foodborne gastrointestinal bacterial infections worldwide. The human disease caused by *Campylobacter*, namely campylobacteriosis, is mostly due to the Gram-negative spiral-shaped *C. jejuni* [1]. This foodborne disease is characterized by reported symptoms including fever, abdominal cramps, bloody diarrhea, dizziness and myalgia [2]. Although such infections tend to be self-limiting, syndromes such as Guillain-Barré and Miller Fisher can be late-onset complications [3]. This enteric pathogen is also a suspected etiological factor in Crohn's disease and ulcerative colitis [1], [4]. *C. jejuni* is one of the principal causes of hospitalization for foodborne illness in the USA [5]. In a comparison of 168 pathogen-food combinations for 14 leading pathogens across 12 food categories representing over 95% of the annual illnesses and hospitalizations in the USA, the combination *Campylobacter*-poultry reached the first rank in terms of annual disease burden including illness, hospitalizations, deaths and costs [6]. A baseline survey conducted in 28 European countries also indicated that the prevalence of *Campylobacter*-colonized broiler batches and *Campylobacter*-contaminated broiler carcasses was 71.2% and 75.8%, respectively [7] which constitutes the main reservoir for human campylobacteriosis. Although this obligate microaerobic pathogen has fastidious growth requirements [8], *C. jejuni* can survive, paradoxically, in food products challenging food processing, conservation and preparation conditions [9]. During these processes, *C. jejuni* is exposed to highly variable oxygen concentrations suggesting that it must develop protective mechanisms to resist oxidative stress [10]. Oxidative stress leads to the degradation and modulation of protein functions and results in lipid and DNA damage [11]–[13]. Kaakoush *et al.* (2007) [14] have shown that *C. jejuni* strains have different oxygen tolerances. A cross-protection between low temperature and oxidative stress in *C. jejuni* strains from various origins has been reported by Garénaux *et al.* (2008) [15]. *Campylobacter* is probably also inactivated by an oxidative burst when high pressure treatment is applied [16]. Moreover, oxidative stress and redox-related proteins were found to be over-expressed in *C. jejuni* stressed with paraquat, a strong oxidizing agent [17].

In *Campylobacter spp.*, oxygen is required as a terminal electron acceptor for respiration [18] and the genes described in other Gram-negative bacteria for oxidative stress and general stress responses are lacking [19], [20]. *C. jejuni* encodes only a few enzymes in oxidative defense, including a superoxide dismutase (SodB), an alkyl hydroperoxide reductase (AhpC) and a catalase (KatA) [21]–[23] for which the molecular and gene regulation mechanisms are still poorly understood [24]. *C. jejuni*, unlike other foodborne pathogens, lacks the key regulators of oxidative stress defense enzymes known in *E. coli* and *S. typhimurium* as SoxRS and OxyR regulons [25]. However, it has been shown that alternative regulators, termed Fur and PerR, mediate at least part of the response to oxidative stress in *Campylobacter* by repressing both AhpC and KatA expression [22], [26]. More recently, two other regulators have been found to be involved in the oxidative stress response [15], [27], [28]. *C. jejuni* also encodes other antioxidant enzymes, such as the thiolperoxidases (Tpx) and the bacterioferritin co-migratory protein (Bcp), which together play a role in the protection of *C. jejuni* against oxidative stress [29], [30]. Hofreuter *et al.* [31] have also indicated that the strain *C. jejuni* 81–176 has an additional DMSO reductase system which may be important for respiration in oxygen-restricted conditions. Respiration is a reactive oxygen species (ROS)-generating process initiated in the microbial membrane. However, no overall approach has yet been used to identify *C. jejuni* membrane proteins involved in the response to oxidative conditions.

As the membrane is the first bacterial line of defense against environmental stresses, proteomic analyses at the membrane level of *C. jejuni* in oxygen-enriched conditions were explored. In the present study, *C. jejuni* inner and outer membrane subproteomes were characterized using two-dimensional protein electrophoresis (2-DE) on oxygen-acclimated cells and oxygen non-acclimated cells and were related to the capability of *C. jejuni* to adhere to abiotic surfaces.

## Results

### *C. jejuni* 81–176 and NCTC 11168 under oxygen acclimation conditions

As *C. jejuni* 81–176 and NCTC 11168 could not survive in atmospheric air, a specific gas mixture was used to explore its oxygen acclimation response. Oxygen acclimation was performed using the same gas mixture for optimal growth (5% O_2_, 10% CO_2_ and 85% N_2_) but with a higher oxygen concentration (19% O_2_, 10% CO_2_ and 71% N_2_) on growing cells. The presence of blood did not change the colony-forming capability of for both strains. This is not surprising as red blood corpuscles contained in blood are able to capture dioxygen. On blood-free plates, almost twice as much time was necessary for the development of *C. jejuni 81–176* colonies under oxygen-acclimation conditions compared to microaerobic conditions while the same number of colonies could not be reached by NCTC11168 in these conditions (Table 1). Consequently, *C. jejuni* 81–176 strain was used for further experiments. An identical number of colonies in both conditions was retrieved for subsequent proteomic analyses of each of the membranes of *C. jejuni*.

**Table 1:** Time required to reach at least 250 colonies in microaerobic and oxygen enriched conditions for three strains of *C. jejuni* grown on Columbia blood agar plates (CBA) and Columbia blood-free agar (CA) plates from a 100 µL spread inoculum of a 10^5^ diluted culture.

		*C. jejuni* NCTC 11168	*C. jejuni* 81–176
**CBA**	5% O_2_, 10% CO_2_, 85% N_2_	15 h	15 h
	19% O_2_, 10% CO_2_, 71% N_2_	15 h	15 h
**CA**	5% O_2_, 10% CO_2_, 85% N_2_	24 h	24 h
	19% O_2_, 10% CO_2_, 71% N_2_	>63 h	42 h

### Separation of membrane proteins of *C. jejuni* 81–176

**Figure 1:**
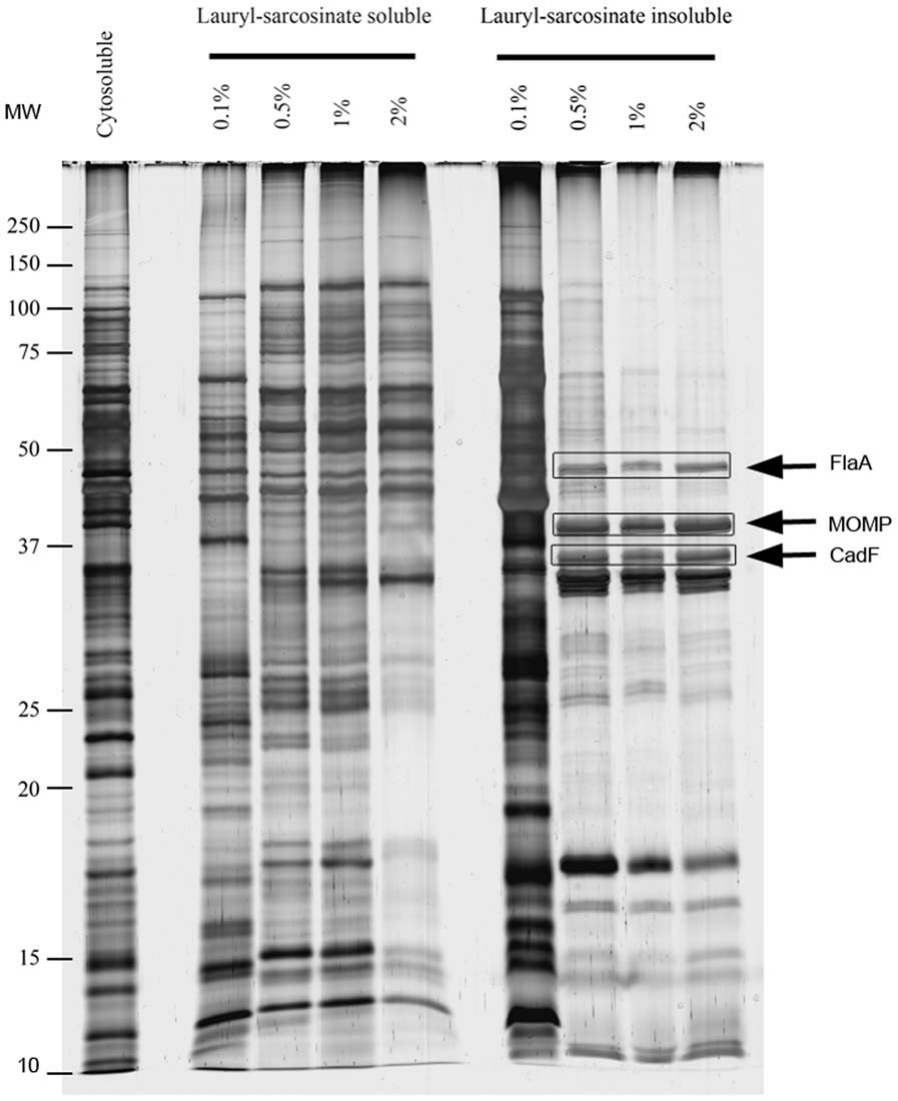
Membrane protein fractions of *C. jejuni* 81–176 extracted with lauryl sarcosinate at 0.1, 0.5, 1 and 2% concentrations and separated using SDS-PAGE. Inner membrane protein-enriched fraction (lauryl-sarcosinate-soluble fraction), outer membrane protein-enriched fraction (lauryl-sarcosinate-insoluble fraction) and cytosolic protein (cytosoluble) profiles are presented. Molecular masses (MM) are indicated on the left (kDa). Identified proteins in the sarcosinate-insoluble fraction are indicated on the right.

Analyzing membrane proteins using 2-DE is complex due to the difficulty in extracting and solubilizing the inherently hydrophobic proteins [32]. The most efficient and reproducible membrane separation for *Campylobacter* was obtained using the method based on lauryl-sarcosinate detergent rather than sucrose density gradient ultracentrifugation or spheroplasting by lysozyme (data not shown) as already observed previously [33]–[35]. The lauryl-sarcosinate activity enables two fractions to be obtained, a lauryl-sarcosinate-insoluble fraction enriched in outer membrane proteins (OMPs) and a lauryl-sarcosinate-soluble fraction enriched in inner membrane proteins (IMPs). As different concentrations of lauryl sarcosinate were previously used on *C. jejuni* (0.2% in Asakura *et al.* [33], 1% in Hobb *et al.* [35] and 2% in Leon-Kempis *et al.* [34]), the lowest efficient concentration was determined to prevent any interference during protein electrofocalization (Fig. 1). From a 0.5% detergent concentration, outer and inner membrane profiles were clearly distinguished and the identification of the main OMPs of *C. jejuni* in the lauryl-sarcosinate-insoluble fraction (FlaA, PorA-MOMP and CadF) confirmed the expected IMP-OMP separation. The lowest concentration of lauryl sarcosinate required to obtain the optimal separation of IMPs and OMPs was thus selected for subsequent 2D-electrophoresis experiments to prevent interference during protein electrofocalisation.

### Membrane subproteome variations in oxygen acclimation conditions

**Figure 2:**
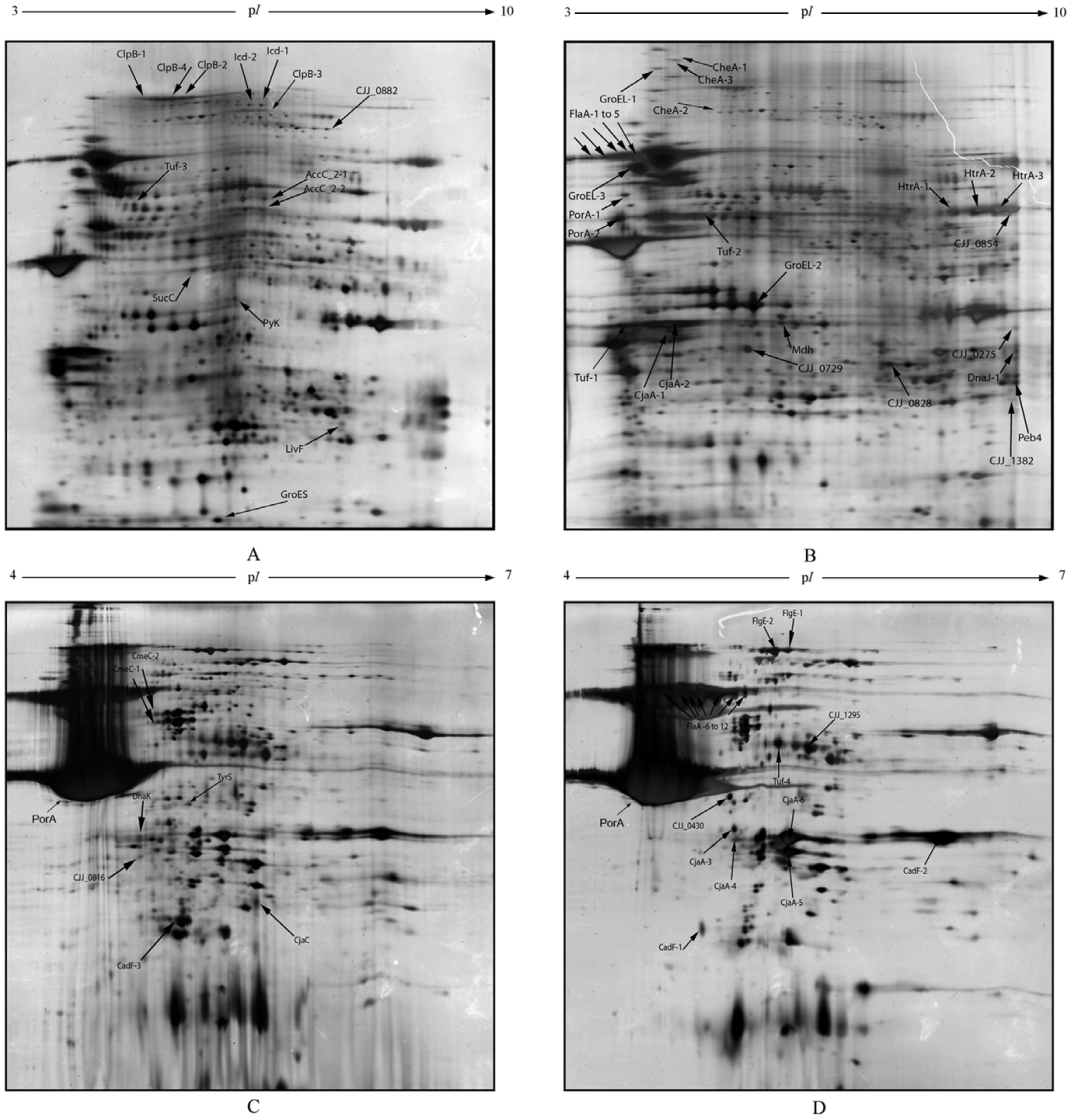
Two-dimensional electrophoresis (2-DE) profiles of the inner (A, B) and outer membrane proteins (C, D) of oxygen-acclimated cells (B, D) compared to non-acclimated cells (A, C) of *C. jejuni* 81–176. On profiles A and C, arrows indicate the significant lower-abundance proteins (or protein forms) in oxygen-acclimated cells while on profiles B and D, arrows indicate the significant higher-abundance proteins (or protein forms) in oxygen-acclimated cells. PorA was identified as the major protein on OMP profiles.

The differences between the 2D-electrophoretic profiles obtained from oxygen-acclimated cells and microaerobically grown cells were validated by PCA (*cf.* Fig. S1). Then, LC-MS/MS analyses successfully identified 42 and 25 spots which exhibited a significantly altered abundance in the IMP-enriched fraction and in the OMP-enriched fraction, respectively (Fig. 2, Table 2). Several of these spots contained the same protein (*e.g.* the FlaA protein with 12 p*I* variants or CadF with 3 p*I* variants). The same observation was made previously on the whole envelope of *C. jejuni* JHH1 studied by Cordwell *et al.* [36]. Finally, a total of 23 higher-abundance proteins and 15 lower-abundance proteins in oxygen-acclimated cells as compared to the control were identified. The localization of these proteins in their cellular compartment was predicted using the algorithm PSORTb v.3.0.2 (Table 2). PSORTb returns a list of the five localization sites for Gram-negative bacteria (cytoplasm, inner membrane, periplasm, outer membrane and extracellular space) and the associated probability value for each. Several proteins were predicted in the cytoplasmic compartment and could thus be regarded as contaminant proteins. This was expected as methods used for membrane fractionation do not separate exclusively membrane proteins [37], [38]. In fact, some of the predicted cytoplasmic proteins have already been described in membrane analysis such as the chaperone proteins GroEL, DnaJ, DnaK, [39] or the elongation factor EF-Tu [39]–[43]. Apart from the localization of intrinsic or secreted proteins being well predicted by the PSORTb algorithm due to their specific structure (signal peptide, transmembrane alpha helices, beta-barrel proteins, hydrophobicity, motif), the extrinsic proteins associated with the surface of the membranes could not be so easily predicted. This could also explain why some of the proteins predicted in the cytoplasm were found in the enriched membrane protein fractions. To avoid any experimental or prediction biases, only proteins isolated from membrane enriched fractions predicted as membrane, periplasmic or secreted proteins were discussed further. The MOMP represents the major part of proteins in the OMP-enriched fraction, as already reported in previous studies [36]. The over-abundance of one protein could prevent the detection of less abundant proteins. Analyzing the two membranes of *C. jejuni* separately reduced this bias in the IMP-enriched fraction while emphasizing it in the OMP-enriched fraction.

**Table 2:** Identification and localization prediction (PSORTb v3.0.2) of proteins predominantly modulated by oxygen-acclimated conditions in the IMP and OMP enriched fractions of *C. jejuni*.

Accession number	Protein ID	Prot/Spot	*P*-value*	n-Fold	p*I/*MW	Mascot score	NMP/Pc	Fraction localization	Cell localization prediction
	***Transporters***								
gi|121612178	CjaA protein	CjaA-1	0.003	up2.0	5.69/30949	799	29/66%	IM	PP/IM
	putative amino acid	CjaA-2	0.009	up2.2	5.69/30949	699	22/65%	IM	PP/IM
	transporter	CjaA-3	0.002	up1.9	5.69/30949	339	14/55%	OM	PP/IM
	[surface antigen CjaA]	CjaA-4	0.008	up2.4	5.69/30949	358	13/54%	OM	PP/IM
		CjaA-5	0.006	up1.6	5.69/30949	865	32/75%	OM	PP/IM
		CjaA-6	0.005	up2.0	5.69/30949	485	18/55%	OM	PPIM
gi|121612379	histidine-binding protein HisJ (surface antigen CjaC) CjaC protein	CjaC		do2.4	6.48/27781	376	10/34%	OM	PP
gi|121613528	high affinity branched-chain amino acid ABC transporter, ATP-binding protein	LivF	0.002	do2.3	7.03/25739	510	16/58%	IM	CytP
gi|121612467	outer membrane lipoprotein CmeC RND efflux system,	CmeC-1	0.01	do1.7	5.14/55385	598	13/29%	OM	OM
		CmeC-2	0.003	do1.9	5.14/55385	526	12/27%	OM	OM
	***Chaperones***								
gi|121612249	chaperonin GroEL	GroEL-1	0.0001	up3.3	5.02/57934	945	27/47%	IM	CytP
		GroEL-2	0.007	up1.6	5.02/57934	2350	68/44%	IM	CytP
		GroEL-3	0.005	up1.8	5.02/57934	1724	45/55%	IM	CytP
gi|121612930	co-chaperonin GroES	GroES	0.004	do2.2	5.38/9452	348	12/87%	IM	CytP
gi|121613084	molecular chaperone DnaK	DnaK	0.005	do2.5	4.98/67403	206	6/12%	OM	CytP
gi|121612573	co-chaperone protein DnaJ	DnaJ1	0.003	up9.0	8.86/33320	79	3/10%	IM	CytP
gi|121613623	ATP-dependent chaperone protein ClpB	ClpB-1	0.004	do3.5	5.47/95489	1802	56/56%	IM	CytP
		ClpB-2	0.003	do2.4	5.47/95489	1742	56/52%	IM	CytP
		ClpB-3	0.002	do2.0	5.47/95489	607	19/22%	IM	CytP
		ClpB-4	0.006	do2.4	5.47/95489	2692	88/68%	IM	CytP
	***Fatty acid biosynthesis***								
gi|121612451	biotin carboxylase	AccC_2-1	0.00001	do1.8	6.01/49116	191	7/19%	IM	CytP
		AccC_2-2	0.001	do2.8	6.01/49116	165	5/17%	IM	CytP
gi|121613559	putative lipoprotein	CJJ_0430	0.01	up3.3	5.29/33188	254	9/29%	OM	unknown
	***Adhesion/virulence***								
gi|121612545	flagellin	FlaA-1	0.00003	up2.1	5.61/59507	1518	41/43%	IM	EC
		FlaA-2	0.00006	up2.0	5.61/59507	1180	29/43%	IM	EC
		FlaA-3	0.0003	up2.0	5.61/59507	1694	45/48%	IM	EC
		FlaA-4	0.0007	up1.8	5.61/59507	1131	31/45%	IM	EC
		FlaA-5	0.00001	up2.1	5.61/59507	1426	34/40%	IM	EC
		FlaA-6	0.00013	up1.8	5.61/59507	1383	33/46%	OM	EC
		FlaA-7	0.0004	up2.7	5.61/59507	601	15/36%	OM	EC
		FlaA-8	0.0007	up1.9	5.61/59507	1884	53/57%	OM	EC
		FlaA-9	0.002	up2.3	5.61/59507	253	10/24%	OM	EC
		FlaA-10	0.003	up2.0	5.61/59507	402	19/33%	OM	EC
		FlaA-11	0.001	up2.4	5.61/59507	2379	67/60%	OM	EC
		FlaA-12	0.0003	up3.3	5.61/59507	1999	46/53%	OM	EC
gi|121613214	flagellar hook protein FlgE	FlgE-1	0.005	up1.7	5.14/89392	915	30/39%	OM	EC
		FlgE-2	0.0006	up1.6	5.14/89392	1320	36/37%	OM	EC
gi|121613274	chemotaxis histidine kinase CheA	CheA-1	0.005	up2.4	4.88/85168	889	20/28%	IM	CytP
		CheA-2	0.006	up2.2	4.88/85168	370	11/15%	IM	CytP
		CheA-3	0.0006	up2.7	4.88/85168	451	13/19%	IM	CytP
gi|121612668	major outer membrane protein (MOMP)	PorA-1	0.002	up2.9	4.72/45681	281	8/20%	IM	OM
		PorA-2	0.002	up1.7	4.72/45681	616	14/38%	IM	OM
gi|121612905	cell-binding factor 2 major antigenic peptide Peb4 CBF2	Cbf2 (Peb4)	0.00003	up5.1	9.23/30411	479	19/51%	IM	PP
gi|121612147	fibronectin-binding protein	CadF-1	0.004	up1.7	5.89/35967	202	6/12%	OM	OM
		CadF-2	0.005	up1.6	5.89/35967	540	13/34%	OM	OM
		CadF-3	0.006	do1.8	5.89/35967	235	6/16%	OM	OM
gi|121613534	fibronectin type III domain-containing protein	CJJ_1295	0.0007	up1.6	5.91/46079	511	19/42%	OM	unknown
	***Other***								
gi|121612430	elongation factor Tu	Tuf-1	0.005	up1.7	5.11/43566	918	28/48%	IM	CytP
		Tuf-2	0.009	up1.6	5.11/43566	2361	65/75%	IM	CytP
		Tuf-3	0.004	do2.2	5.11/43566	570	15/36%	IM	CytP
		Tuf-4	0.001	up1.7	5.11/43566	992	31/68%	OM	CytP
gi|121613042	serine protease DO	HtrA-1	0.006	up1.6	8.97/50985	1515	44/61%	IM	PP
		HtrA-2	0.009	up1.7	8.97/50985	1626	50/62%	IM	PP
		HtrA-3	0.00007	up4.3	8.97/50985	475	15/31%	IM	PP
gi|121613659	malate dehydrogenase	Mdh	0.002	up2.0	5.46/33379	212	5/19%	IM	CytP
gi|121612371	succinyl-CoA synthase, beta subunit	SucC	0.006	do1.7	5.61/41716	164	6/16%	IM	CytP
gi|121612541	isocitrate dehydrogenase, NADP-dependent	Icd-1	0.004	do2.9	6.85/86316	156	5/9%	IM	CytP
		Icd-2	0.007	do2.1	6.85/86316	954	27/36%	IM	CytP
gi|121612912	pyruvate kinase	Pyk	0.005	do1.8	5.89/53751	485	16/31%	IM	CytP
gi|121612884	arylsulfate sulfotransferase, degenerate	CJJ_0882	0.005	do1.8	7.57/69255	411	15/27%	IM	unknown
gi|121613032	mur ligase family protein	CJJ_0816	0.01	do2.3	9.22/55168	34	2/4%	OM	unknown
gi|121613329	tyrosyl-tRNA synthetase	TyrS	0.0005	do3.4	6.31/45347	31	5/16%	OM	CytP
gi|121613150	hypothetical protein CJJ81176_1382	CJJ_1382	0.00005	up3.5	8.84/26552	143	4/23%	IM	OM/PP/EC
gi|121613455	hypothetical protein CJJ81176_0729	CJJ_0729	0.006	up1.6	5.60/27722	795	23/67%	IM	CytP
gi|121613526	7-alpha-hydroxysteroid dehydrogenase	CJJ_0828	0.001	up1.8	6.77/28119	1025	35/88%	IM	CytP
*gi|121612484*	*hypothetical protein CJJ81176_0854*	*CJJ_0854*	0.007	up7.3	5.70/36925	16	3/11%	IM	CytP
*gi|121612647*	*hypothetical protein CJJ81176_0275*	*CJJ_0275*	0.002	up7.8	5.32/31916	16	3/13%	IM	unknown

* Only spots with a *q*-value (False Discovery Rate) <0.05 and a P (Power)>0.8 were conserved.

Spot refers to spots detected from 2-DE gel analysis (Fig. 2A and B), N-fold: protein abundance difference between 5% O_2_ and 19% O_2_, up: higher abundance protein, do: lower abundance protein, p*I*: protein isoelectric point; MW: protein molecular weight (Da); NMP: number of matching peptides, Pc: % of protein coverage, OM: outer membrane, IM: inner membrane; PP: periplasm, EC: extracellular, CytP: cytosol. The identification of the proteins indicated in italic was not statistically validated.

Among the 38 identified proteins whose abundance was modulated by oxidative conditions, four main functional classes were described : (i) transporters that could be involved in the setting up of new catabolic pathways (CjaA/CjaC, LivF, CmeC), (ii) chaperones in response to the oxidative stress (DnaK, GroEL/S, DnaJ1, ClpB), (iii) proteins involved in fatty acid biosynthesis (AccC) and (iv) proteins involved in the adhesion/virulence of *C. jejuni* 81–176 (FlaA, FlgE, CadF, Cjj_1295, Peb4, CheA, MOMP).

### qRT-PCR analysis of proteins identified in 2-DE

**Figure 3:**
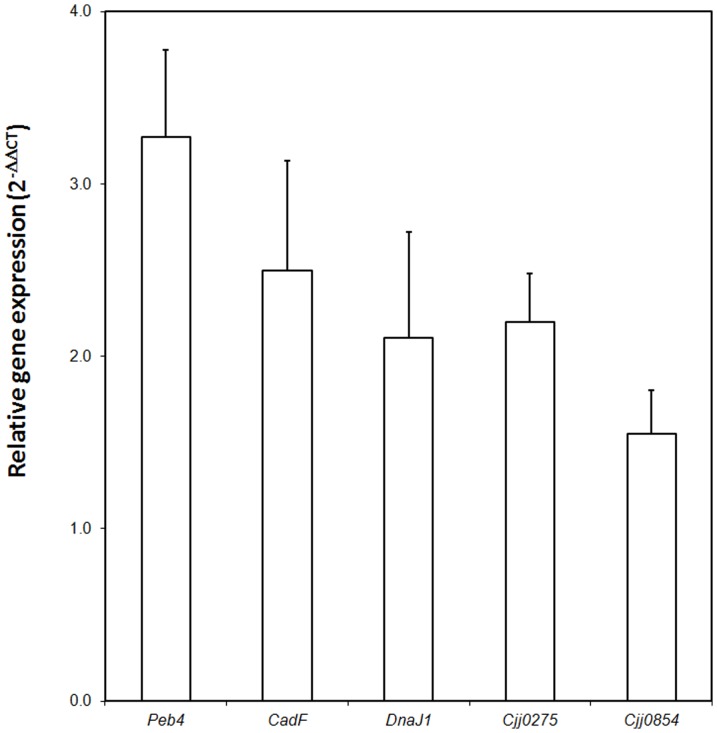
Relative mRNA levels of *peb4*, *cadF*, *dnaJ*, *cjj0275* and *cjj0854* as revealed by qRT-PCR in oxygen-acclimated *C. jejuni* 81–176 normalized to relative mRNA levels observed in non-acclimated cells (equivalent to 1). The *rpo*A gene was used as the endogenous control. Error bars represent the standard deviation of the mean of three independent RNA extractions. Significant differences between oxygen-acclimated and non-acclimated cells were validated statistically (0.002<*P*<0.035).

Differently expressed proteins of interest in oxygen-acclimated cells were selected to further investigate their expression patterns at the transcription level (Fig. 3). The selection was based on the most over-expressed proteins *i.e.* above 5.0-fold: the major antigenic peptide Peb4 (5.1-fold), the co-chaperone DnaJ1 (9-fold), Cjj_0854 (7.3-fold) and Cjj_0275 (7.8-fold). Although the identification of two hypothetical proteins could not be statistically validated, they were also selected to assess their expression profile in oxygen-acclimated cells. The protein CadF was selected too as its abundance among cytosoluble proteins has previously been reported as being modulated under paraquat-mediated oxidative stress [15]. The qRT-PCR results indicated that gene expression patterns of the proteins were in accordance with the proteomic-level changes for all proteins. All the genes tested were significantly more transcribed in oxygen-acclimated cells (*P*<0.05). However, the relative mRNA expression level was not proportional to the level of protein abundance for all the genes. For instance, Cjj_0854 displayed the lowest mRNA expression while the fold was one of the highest recorded (7.3-fold). This may be attributed to the relative stability of the mRNA and proteins or to the differences in regulation mechanisms (such as degradation rates and protein synthesis) that act on both mRNA synthesis and protein synthesis, and ultimately affect the combined molecular amounts [44].

### Swarming capability of oxygen-acclimated cells

**Figure 4:**
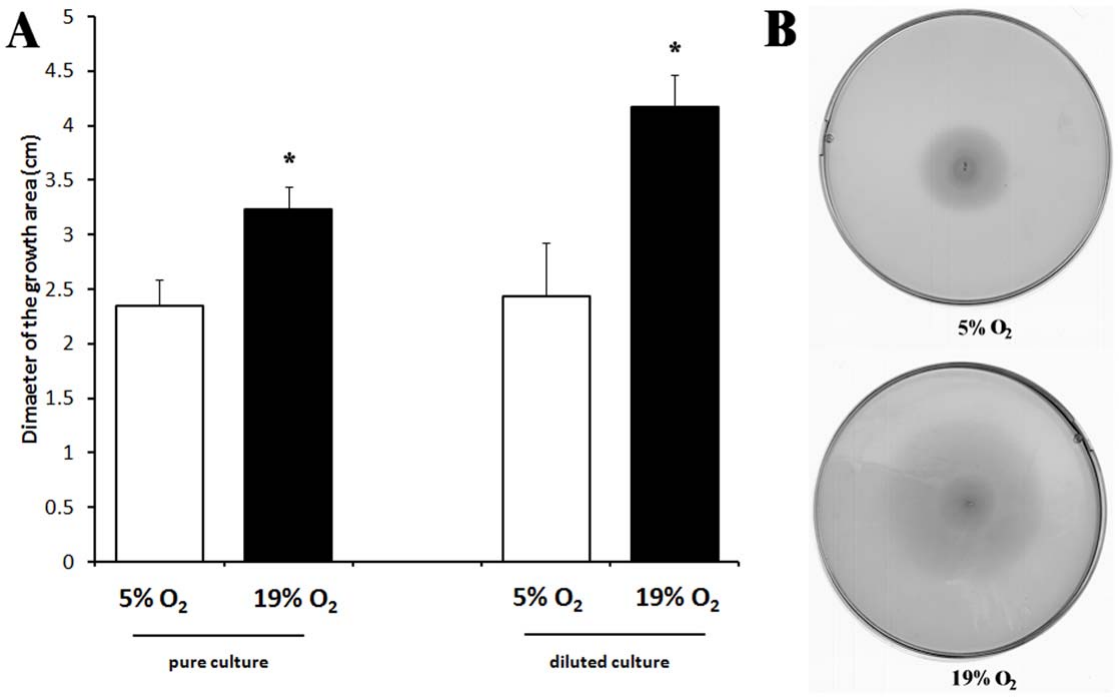
Motility of *C. jejuni* 81–176 on BHI+0.6% agar in microaerobic (5% O_2_) and oxygen-enriched conditions (19% O_2_) after 48 h at 42°C. Assays were performed with 2 µL of pure culture or 10 times diluted culture. (A) Mean diameters of three independent experiments. (B) Example of motility plates obtained after 48 h at 42°C with a diluted culture.

As flagellum components (FlaA and FlgE) were over-expressed in oxygen-acclimated conditions, the swarming capability of *C. jejuni* 81–176 was assessed in optimal growth conditions and in oxygen-acclimated conditions (Fig. 4). After 48 h incubation on soft agar, the results revealed that swarming capability was significantly enhanced in oxygen-acclimated conditions as compared to microaerobic conditions.

### Adhesion of oxidative-stressed cells and oxygen-acclimated cells to abiotic surfaces

**Figure 5:**
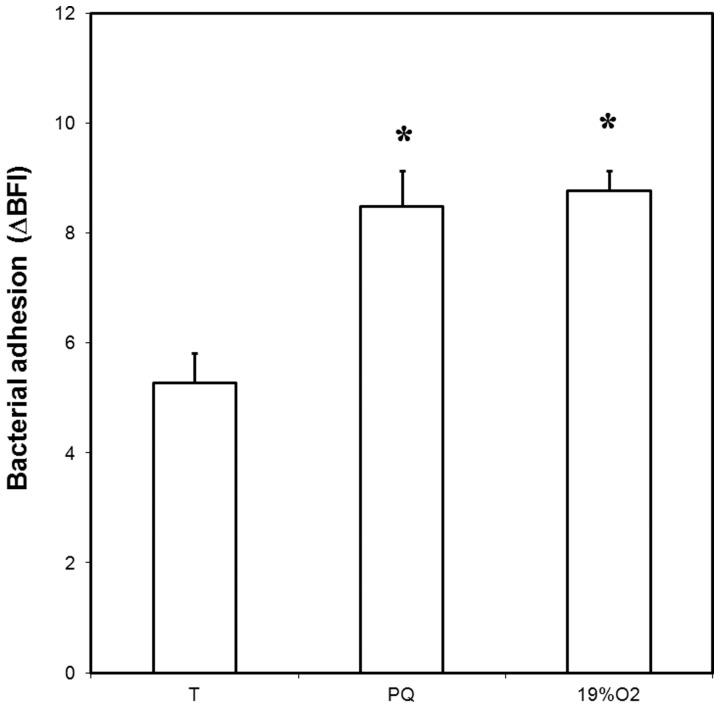
Adhesion capability after 0.5 h to an inert surface of oxygen-acclimated cells and oxidative-stressed cells of *C. jejuni* 81–176. 19% O_2_: oxygen-acclimated cells, (PQ) oxidative- stressed cells mediated by paraquat, (T) control without oxidizing agents (microaerobic conditions). Bacterial initial concentration was 8.81±0.05 Log(CFU/mL) for oxygen-acclimated cells, 8.20±0.11 Log(CFU/mL) for PQ-stressed cells and 8.85±0.05 Log(CFU/mL) for the control. Error bars represent the standard deviation of three independent assays. Asterisks indicate significant differences (*P*<0.05) in comparison with the control.

The capability of oxygen-acclimated cells as well as paraquat-stressed cells to adhere to an inert surface was estimated using the Biofilm Ring Test® (Fig. 5). This test was designed to assess both bacterial adhesion and biofilm formation in 96-well microtiter plates. The test is based on the reduced detection of magnetic beads entrapped by the adherent bacterial cells. It has been applied to various bacteria able to adhere to inert surfaces (e.g. [45]–[47]) and found especially appropriate for assessing *Campylobacter* adhesion [48]. As *C. jejuni* 81–176 adhesion is close to the detection limit using the Biofilm Ring Test® after 2 h, any effect that would increase the number of adherent cells could not be correctly assessed. For this reason, the adhesion experiments were performed after 0.5 h when fewer adherent cells in the control under microaerobic conditions were detected [48]. Paraquat, a superoxide anion generator, was used to induce an oxidative stress as previously described [15] on broth cultivated cells. Cells stimulated by paraquat or acclimated to enriched oxygen conditions displayed a greater adhesion capability than those cultivated in microaerobic conditions, indicating that oxidizing agents have an impact on the very first step of biofilm development. No significant difference was observed between oxygen-acclimated cells and paraquat-stressed cells.

### Identification of CadF protein forms using immunoblotting

**Figure 6:**
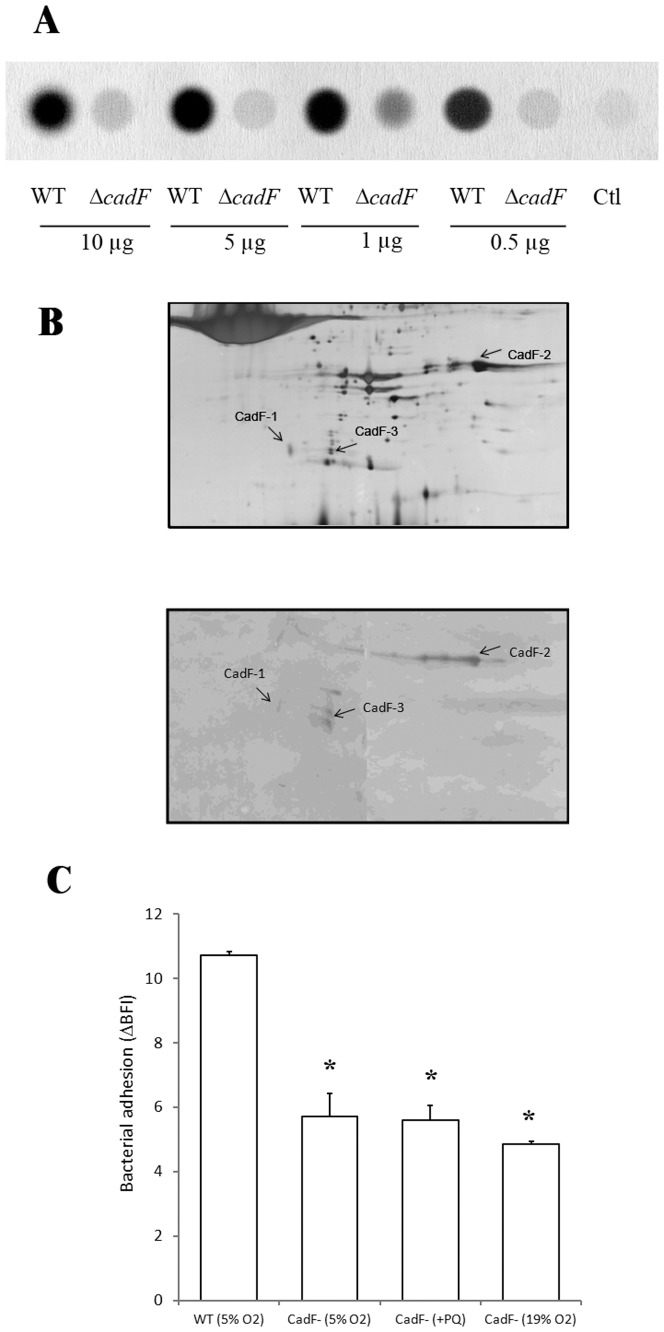
Influence of CadF on *C. jejuni* adhesion to inert surfaces. (A) Dot blotting using anti-CadF antibodies of 0.5, 1, 5 and 10 µg of OMP-enriched fraction of *C. jejuni* 81–176 (WT) and the derivative mutant *C. jejuni* 81–176 Δ*cadF*. Ctl is the control without protein. (B) Silver-stain two-dimensional electrophoretic gel of the OMP-enriched fraction of oxygen-acclimated cells and the corresponding immunoblot using anti-CadF antibodies. (C) Adhesion capability after 2 h of oxygen-acclimated cells and paraquat-stressed cells of *C. jejuni* 81–176 mutant Δ*CadF* (CadF^−^) and its isogenic strain. Initial bacterial concentration anged from 8.72 to 8.85 Log(CFU/mL). Error bars represent the standard deviation of three independent assays. Asterisks indicate significant differences in comparison with the isogenic strain (*P*<0.05).

The absence of CadF protein in the OMP enriched fraction of the derivative 81–176 Δ*cadF* mutant as compared to the isogenic strain was verified using dotblotting (Fig. 6A). The immunoblot using anti-CadF antibodies performed from the 2-DE gel of the sarcosyl-insoluble fraction of oxygen-acclimated cells confirmed the identification of different forms of CadF. These included the two higher-abundance forms (CadF-1 and CadF-2) and the lower-abundance form (CadF-3) in oxygen-acclimated conditions (Fig. 6B).

### Effect of cadF mutation on adhesion to an inert surface of paraquat-stressed cells and oxygen-acclimated cells

The *C. jejuni* 81–176 mutant Δ*cadF* was significantly less adherent than its isogenic strain (Fig. 6C). In addition, neither oxygen-acclimated cells nor paraquat-stressed cells from the mutant recovered their initial adhesion level, confirming that CadF is involved in the adhesion process of *C. jejuni* to inert surfaces.

## Discussion

The purpose of this study was to examine the response of microaerophilic *C. jejuni* 81–176 to oxygen-enriched conditions at the membrane protein level. Oxygen was selected instead of using chemicals generating ROS molecules, such as hydrogen peroxide and paraquat, which are frequently applied to induce oxidative stress in *C. jejuni*. This enabled efflux pump activation to be encompassed such as that reported in *Salmonella enterica* for paraquat efflux [49], [50] and, more recently, in *C. jejuni* with CmeG [51] for oxygen peroxide (H_2_O_2_) efflux. In addition, a single method of growth was chosen to avoid any cellular changes due to the method [52]. To segregate the influence of oxygen from that of any other gas, controlled mixtures of gas essential for *C. jejuni* growth (O_2_, CO_2_) were used. As a capnophilic bacterial species, *C. jejuni* is able to assimilate CO_2_, which could be explained by a reverse reaction producing pyruvate by a *f*lavodoxin *q*uinone *r*eductase FqrB (Cjj_0584) as demonstrated in the closely related species *Helicobacter pylori* [53]. Thus, O_2_ concentration could be varied while the CO_2_ concentration was maintained at a constant level. However, oxygen is a less powerful oxidizing molecule and its solubility is very low in liquid at 42°C (Henry's constant = 9.28×10^−4^ mol L^−1^ atm^−1^ in water at 42°C). In our study, oxygen transfer was reduced by applying controlled gas mixtures to cells forming colonies. The mixture of 19% O_2_, 10% CO_2_ and 71% N_2_ was used to obtain oxygen-acclimated cells while 5% O_2_, 10% CO_2_ and 85% N_2_, which is the modified atmosphere usually applied for optimal growth conditions, was used as a control.

Differential protein expression between these two conditions was analyzed on the IMP-enriched and the OMP-enriched fractions separated using lauryl-sarcosinate as previously applied to *C. jejuni* membrane fractionation [33], [34]. This is the first time that such 2-DE subproteomic analysis has been reported on separate membrane fractions of *Campylobacter*. Our data demonstrated that the adaptation of *C. jejuni* 81–176 to oxygen-enriched growth conditions resulted in the differential abundance of some proteins in both membranes. These could be grouped into four identified functional classes representing transporters, chaperones, adhesion/virulence and fatty acid synthesis. As all the identified membrane-associated proteins related to adhesion/virulence were more abundant in oxygen-acclimated cells, downstream analyses were focused on this functional class.

The higher-abundance membrane proteins FlaA and FlgE in oxygen-acclimated cells are involved in cell motility and subsequently in the virulence of *C. jejuni* as non-motile or motile-restricted cells have been shown to be less virulent [54], [55]. FlaA was found in both membranes and FlgE in the outer membrane and they are both predicted to be secreted. The flagellum comprises a basal body (a conduit spanning the inner and outer membranes of the cell), a hook section of the flagellum composed primarily of the protein FlgE, and the flagellar filament, which consists of thousands of copies of the flagellin proteins FlaA and FlaB, with FlaA being the major component. It was not surprising to detect FlaA in both membranes and for it to be predicted to be secreted as it is exported through the two membranes for filament elongation. *C. jejuni* possesses a flagellum that functions in both motility and protein secretion [56]–[59]. The higher expression of flagellum components is consistent with the increased swarming ability in oxygen enriched conditions. Differences in the swarming motility of *C. jejuni* were also observed using various methods to obtain a microaerobic atmosphere with a concomitant enhanced swarming and a higher transcript level of *flaA* [52]. Over-expression of FlaA has previously been reported in *C. jejuni* NCTC 11168 stressed with paraquat [15] and in a robust colonizer of the chicken gastrointestinal system [60]. Using aflagellate and non-motile mutants inactivated on *maf5* or *fliS* genes [61] or deleted on the *flaAB* gene [62], a severely reduced aggregate biofilm was observed. As in many flagellated bacteria, flagella are involved in *C. jejuni* biofilm formation [63]–[67].

Peb4 was predicted in the periplasm and found in the inner membrane, which is in accordance with the localization previously described [36]. In our study, this protein was induced in oxygen-enriched conditions as revealed by its higher abundance and concomitant increase gene expression. The highly conserved periplasmic Peb4 of *C. jejuni* 81–176 is similar to the peptidyl prolyl cis-trans isomerase (SurA) in *E. coli* and other orthologs in numerous bacteria. It constitutes a major antigen of *C. jejuni* and may be involved in the energy-generation-free transformation of carbohydrates, as well as in the folding of outer membrane proteins [33], [68]. A *peb4* mutant of *C. jejuni* NCTC 11168 [33] and *C. jejuni* 81–176 [69] was reported to impact biofilm formation. In addition, the *peb4* mutant cells of NCTC 11168 impaired the increase in biofilm formation in an ambient air environment suggesting that Peb4 is involved in biofilm formation [33].

No biological function has yet been attributed to Cjj_1295; however, its DNA sequence possesses a fibronectin-type III domain which suggests a possible role in fibronectin recognition. The OMP CadF (*C*ampylobacter *a*dhesin to *F*ibronectin) promotes the binding of *C. jejuni* to fibronectin (*F*n) on host cells [70] and is required for maximal adherence and invasion of INT407 cells and colonization of the chicken cecum [71], [72]. Furthermore, CadF was also found to be more abundant after a paraquat-mediated oxidative stress in the soluble protein fraction of *C. jejuni* NCTC 11168 [15]. The relatively higher gene transcription of *CadF* in oxidative conditions compared to microaerobic conditions indicates that this gene is induced in conditions favoring a net accumulation of ROS.

As some over-expressed proteins have previously been identified as signatures of biofilm formation in *C. jejuni*, adhesion to inert surfaces was compared for oxygen-acclimated cells and microaerobically grown cells. Interestingly, cells acclimated to oxygen-enriched conditions enhanced *C. jejuni* adhesion to inert surfaces. In addition, the comparable adhesion obtained with cells stressed with paraquat confirmed that ROS-generating conditions enhanced adhesion of *C. jejuni* to inert surfaces. Furthermore, examining the effect of oxidative conditions prior to adhesion in our study rather than during biofilm formation indicated that these conditions enhanced the first step of biofilm formation by modifying the cell biology to achieve a better adhesion capability. Subsequently, oxidative conditions confer a survival and dissemination advantage of *C. jejuni* through adhesion to abiotic surfaces in the food environment *sensu lato*.

Although the higher abundance of CadF in the outer membrane in oxygen-enriched conditions was modest, it was statistically validated and corroborated with its higher transcription in these conditions and, as previously shown, its over-expression among cytosoluble proteins in *C. jejuni* NCTC 11168 [15]. Consequently, using an insertional inactivation of the *cadF* gene in *C. jejuni* 81–176, a *cadF* mutant was tested for its capability to adhere to an abiotic surface. The lower adhesion capability of the *cadF* mutant compared to its isogenic strain indicates that CadF also plays a role in the inert surface adhesion process and may contribute to the enhanced adhesion in oxygen-enriched conditions. Furthermore, the adhesion of *CadF* mutant cells submitted to oxidative conditions mediated by both paraquat and oxygen was not different from that of CadF mutant cells cultivated in microaerobic conditions, confirming that CadF is a key protein in the adhesion mechanism of *C. jejuni* to inert surfaces. Taken together, these results suggest that adhesion to inert surfaces is also mediated by CadF whose expression is controlled by oxidative conditions. The alignment of CadF sequences indicates that this protein is well-conserved among *C. jejuni* strains suggesting its functional importance (*cf.* Fig. S2). Three forms of CadF (CadF-1, 24 kDa, CadF-2, 35 kDa, and CadF-3, 25 kDa), also detected by immunoblotting, were modulated under oxygen-enriched conditions. Cordwell *et al.* [36], [73] have also previously observed a series of spots corresponding to CadF on 2-DE gels of the entire membrane of *C. jejuni* with variations in their immunogenic properties. The authors also reported two cleavage sites between serine^195^ and leucine^196^, and glycine^201^ and phenylalanine^202^ (nucleotide counts without the 16 nt-peptide signal). Among the three p*I* variants of CadF modulated by oxygen-enriched conditions in our study, CadF-2 displayed a higher molecular weight (35 kDa/p*I* 6.01) than that of CadF-1 and CadF-3 (24 kDa/p*I* 4.85 and 25 kDa/p*I* 5.07, respectively). Noticeably, the protein coverage of CadF-2 included the cleavage site while peptides for CadF-1 and CadF-3 matched only in the N-terminal region suggesting a cleavage of these proteins (*cf.* Fig. S3). This cleavage could be carried out by the carboxyl-terminal protease and the HtrA serine protease [36]. An increased abundance of HtrA has been associated with robust chicken colonization and may reflect a requirement for protease activity in colonization [60]. Interestingly, HtrA was also more abundant in oxygen-enriched conditions in our study.

In conclusion, these data demonstrate that oxygen-enriched conditions promote over-expression of the membrane proteins involved in the biofilm initiation and virulence of *C. jejuni*. The adhesion of *C. jejuni* to inert surfaces results in a complex process which exacerbates and employs proteic virulence factors. Finally, even though aerobic conditions are detrimental to *C. jejuni* growth, sub-lethal oxidative conditions could favor its survival throughout food processing because it develops a greater ability to adhere to inert surfaces, which could explain the re- and cross-contamination of food products by this pathogen.

## Materials and Methods

### Bacterial strains, media and growth conditions

The clinical *Campylobacter jejuni* strains NCTC 11168, 81–176 and its Δ*cadF* derivative generated via insertion of the kan^r^ cassette were used in this study. A loopful of frozen culture conserved at −80°C in Brain-Heart Infusion (BHI) broth (Biokar, Beauvais, France) containing 20% sterile glycerol was spread on fresh Karmali agar plates (Oxoid, Dardilly, France) and incubated in microaerobic conditions of 5% O_2_, 10% CO_2_ and 85% N_2_ (Air Liquide, Paris, France) at 42°C for 48 h. Then, a subculture was performed in BHI in 24-well plates incubated for 18 h at 42°C under microaerobic conditions (5% O_2_, 10% CO_2_ and 85% N_2_, Air Liquide) or oxygen-acclimated conditions (19% O_2_, 10% CO_2_, and 71% N_2_, Air Liquide) with shaking. Next, calibrated inocula (100 µL of a 10^5^ diluted culture) obtained from the subculture were spread on Columbia blood-free gelose plates (Merck KgaA, Darmstadt, Germany) or Columbia plates supplemented with 5% defibrinated horse blood and incubated at 42°C in stainless steel jars (Don Whitley Scientific Ltd, West Yorkshire, UK) under microaerobic conditions or oxygen-acclimated conditions from 15 to 68 h. Each jar was successively vacuum-emptied and refilled twice before incubation to ensure the correct gas concentration. For *C. jejuni* 81–176, cells were harvested from plates flooded with 5 mL of sterile peptone water and the colonies were removed from the agar plates with a cell scraper after 24 h in optimal growth conditions (microaerobic conditions) and after 42 h in the oxygen-acclimated conditions in order to obtain an equivalent number and size of countable colonies.

Cells oxidatively stressed with paraquat, a molecule generating free radicals [74], were obtained as previously described by Garénaux *et al.* [15] and used afterwards for adhesion assays. Briefly, cells grown in the above-mentioned microaerobic conditions were centrifuged for 20 min at 3000×*g* at 20°C and resuspended up to an optical density (OD) of 1.00±0.05 at 600 nm in sterile peptone water broth (PWB) (Merck, Darmstadt, Germany) containing 500 µM paraquat (MP Biomedicals, Illkirch, France) and then incubated for 1 h at 42°C under microaerobic conditions. A control of non-stressed cells was exposed to the same conditions without paraquat. After incubation, cells were harvested by centrifugation for 20 min at 3000×*g* at 20°C and used for adhesion assays.

### Bacterial adhesion to an abiotic surface

Adhesion was assessed for each strain in microaerobic static conditions using the BioFilm Ring Test® (BioFilm Control, Saint-Beauzire, France) according to the protocol described in detail by Sulaeman *et al.* [48]. Briefly, cultures calibrated to OD_600 nm_ = 1±0.05 were added to 8-well polystyrene strips and incubated for 0.5 or 2 h under microaerobic conditions (5% O_2_, 10% CO_2_ and 85% N_2_) or oxygen enriched conditions (19% O_2_, 10% CO_2_ and 71% N_2_). The adhesion capability of each strain was expressed as the mean of the BioFilm Index (BFI) of three wells calculated by the software. Detection is based on attracted beads forming a black spot in the bottom of the wells detected by the Scan Plate Reader (BioFilm Control). The initial concentration before adhesion was verified by plating the appropriate decimal dilution on Blood Gelose plates (Oxoid, Basingstoke, UK). Colonies were enumerated after incubation in microaerobic conditions at 42°C for 48 h. The initial bacterial concentration was calculated from the mean of colonies enumerated on three plates of the appropriate dilution. Three independent assays (from independent cultures) for each condition were performed.

### Bacterial motility

The swarming motility of *C. jejuni* 81–176 was assessed according to Scott *et al.* (2007) [75] with the following modifications. Swarm 0.6% soft agar BHI plates were briefly dried, inoculated with 2 µL of pure culture or 10 times diluted cultures and incubated at 42°C in microaerobic conditions or oxygen-acclimated conditions for 48 h. After incubation, strain motility was calculated by measuring the diameter of the growth area. The experiments were carried out in triplicate from three independent cultures.

### Membrane protein extraction

After ultrasound treatment of bacterial cells, cytoplasmic proteins were separated from membrane fractions by ultracentrifugation at 188,000×*g* for 1 h at 4°C as described previously in Bièche *et al.* (2011). Then, inner membrane proteins (IMPs) and outer membrane proteins (OMPs) contained in the pellet were separated by a sodium lauryl sarcosinate (Sigma-Aldrich, Steinheim, Germany) treatment [76] carried out on ice for 20 min with shaking. To determine the optimal separation conditions, 0.1, 0.5, 1 and 2% sodium lauryl sarcosinate concentrations were tested and only 0.5% was used subsequently. After ultracentrifugation at 188,000×*g* for 1 h at 4°C, supernatant containing IMPs and pellet containing OMPs resuspended in 1 mM EDTA were aliquoted and stored at −80°C. The protein concentration of OMPs and IMPs was determined using the Micro BCA™ Protein Assay Kit (Perbio Science, Brebieres, France) according to the manufacturer's protocol.

To check the optimal separation of IMPs and OMPs using sodium lauryl sarcosinate, migration through 12.5% acrylamide/bisacrylamide SDS-PAGE (18×20×0.75 mm) was performed with a 4% acrylamide/bisacrylamide stacking gel using a Protean II cell (Bio-Rad, Hercules, CA, USA) SDS-PAGE. Samples of 10 µg of proteins were mixed in a ratio of 3∶1 with the reducing agent sample buffer containing 2% SDS, 62.5 mM Tris-HCl, pH 6.8, 10% glycerol, 5% β-mercaptoethanol, and a trace of bromophenol blue, and heated for 5 min at 100°C. Proteins in gels were silver stained and scanned with a GS-800 densitometer (Bio-Rad) operated with the QuantityOne® software (Bio-Rad) at a resolution of 42.3 microns.

### Two-dimensional gel electrophoresis (2-DE)

A quantity of 100 µg of protein was concentrated using the Concentrator 5301 (Eppendorf, Le Pecq, France), at room temperature, until the solution volume was reduced to 40 µL. Then, each sample was solubilized in 400 µL of a solution containing 7 M urea, 2 M thiourea, 2 mM tributyl phosphine (TBP), 1% amidosulfobetaine-14 (ASB-14), 2% carrier ampholytes and 0.25% Coomassie Blue R-250 (Sigma-Aldrich) under rotation shaking for 1 h. Next, each protein sample was frozen/thawed for at least 30 min at −80°C. Insoluble material was removed by centrifugation at 8000×g for 3 min at 4°C. Iso-Electro Focusing (IEF) (Bio-Rad, Marnes la Coquette, France) was carried out with 18-cm NonLinear Immobiline Dry IPG strips (GE Healthcare, Uppsala, Sweden). Gradients giving the best results in terms of spot quantity, quality and separation were used: a linear gradient pH 4–7 for OMPs and a non-linear gradient pH 3–10 for IMPs. Strips were rehydrated under 50 V for 10 h and the voltage for protein focalization was as follows: 250 V for 15 min, a linear increase to 10,000 V for 3 h, then 10,000 V until 105 kVh was reached. After focalization, IPG strips were first reduced for 10 min in 2% dithiothreitol (DTT) and subsequently alkylated for 10 min in 2% iodoacetamide, contained in a detergent exchange buffer composed of 7 M urea, 50 mM Tris-HCl (pH 6.8), 15% glycerol, 2% SDS and 1% Coomassie Blue R-250 as described previously by Vilain *et al.* [77].

The second-dimension electrophoresis was performed using 12.5% acrylamide/bisacrylamide SDS-PAGE. Strips were laid over the separation gel and embedded with 0.3% migration buffer supplemented with 5% polyacrylamide alignment gel. Migration was carried out at 4°C at 300 V maximum and 10 mA/gel for 45–60 min and then at 20 mA/gel. For protein identification, gels were loaded with 250 µg of proteins and stained in 0.06% Colloidal Coomassie Blue G-250 (Bio-Rad). All experiments were carried out in triplicate from three independent protein extractions.

### Image and statistical analyses of 2-DE gels

After scanning by the ProXPRESS Proteomic Imaging System (Perkin Elmer) with a resolution of 100 microns, the 2D gels were analyzed using Progenesis Samespots® 4.0 software (NonLinear Dynamics, Newcastle upon Tyne, UK) for normalization of the gels and statistical analysis. In Progenesis SameSpots, normalization is performed from a calculated gain factor including variations from sample quantity to scanning setting and gel staining which avoid any variations due to major proteins. The quality of the gels was ensured using the Quality Control (QC) of Progenesis SameSpots software. Statistical analysis of protein expression was performed on at least six gels for each condition (5% O_2_ and 19% O_2_), *i.e.* 3 independent experiments (independent cultures) and at least 2 technical replicates for each independent culture. Differences between each condition were validated by Principal Component Analysis (PCA) to determine if samples had the groupings expected or if there were any outliers in the data. In our study, only normalized spots exhibiting variations with a fold of at least 1.6 and with a *p-value* (ANOVA) ≤0.01, a *q-value* (False Discovery Rate, FDR) <0.05 and P (Power Analysis) >0.8 were selected as being differentially expressed. P depends on the sample size and can calculate the effect of running a different number of replicates. With a target power of 0.8, it is possible to select a fold of at least 1.6 without increasing the risk of including false positive spots.

### In-gel trypsin digestion and protein identification

Manually excised spots were washed several times with water and ammonium carbonate, dehydrated with acetonitrile (ACN) and dried. Trypsin digestion was performed overnight with a dedicated automated system (MultiPROBE II, PerkinElmer). The gel fragments were subsequently incubated twice in an H_2_O/ACN solution for 15 min, then in 1% (v/v) Formic Acid for 15 min and finally in 100% ACN for 15 min to enable the extraction of peptides from the gel pieces. Supernatants were pooled and transferred into a clean 96-well plate. Peptide extracts were then dried and solubilized in 10 µL starting buffer for chromatographic elution, consisting of 3% ACN and 0.1% HCOOH in water.

Peptides were analyzed using a nano-LC1200 system coupled to a 6340 Ion Trap mass spectrometer equipped with an HPLC-chip cube interface (Agilent Technologies, Massy, France). The tandem mass spectrometry peak lists were extracted using the DataAnalysis program (version 3.4, Bruker Daltonic) and compared to the *C. jejuni*, strain 81–176, amino acid sequence database (UniprotKB, 09.09.2010) using the Mascot Daemon (version 2.1.3) search engine. The searches were performed with a maximum of one missed cleavage, with no fixed modification and with variable modifications for carbamidomethyl and oxidation of methionines. Identification from the tandem mass spectrometry spectra was performed with a mass tolerance of 1.6 Da for precursor ions and 0.8 for MS/MS fragments. The determination of at least two peptide sequences with a Mascot Score over 50 using splitting patterns allowed a satisfactory identification of the protein. Cell localization was predicted using PSORTb v3.0.2 programs (http://www.psort.org/psortb/) [78].

### Dot and western blotting

Dot blotting was carried out by slowly spotting 5 µL of 0.5, 1, 5 and 10 µg of OMP-enriched fraction onto a nitrocellulose membrane. Western blotting was performed on proteins separated on the unstained 2-DE gel of the OMP-enriched fraction. Proteins were transferred (at 100 V for 2 h) to a nitrocellulose membrane using the Mini Trans-Blot Cell Assembly® SD Semi-dry Electrophoretic Transfer Cell (Bio-Rad).

Non specific sites were blocked by soaking each nitrocellulose membrane for 2 h in 20 mM Tris-HCl, 150 mM NaCl-pH 7.5 supplemented with 4% skim milk. Transferred proteins were probed with a 1/2000 dilution of rabbit anti-serum anti-CadF. Immunoreactive proteins were detected using a 1/2000 dilution goat-anti-rabbit alkaline phosphatase antibody, followed by incubation in 3,3′-diaminobenzidine tetrahydrochloride (DAB) (Sigma-Aldrich). Gels were scanned using a GS-800 Imaging densitometer (Bio-Rad).

### RNA extraction and quantitative RT-PCR (qRT-PCR)

Cell cultures supplemented with 1 mL RNA Protect Reagent (Qiagen, Courtaboeuf, France) were pelleted (3300 *g*, 6 min) at 4°C and resuspended in 1 ml of EXTRACT-ALL® (Eurobio, Courtaboeuf, France) according to the manufacturer's instructions. Then, the RNA samples were treated and submitted to reverse transcription according to Ritz *et al.* (2009) [79]. Quantitative real-time PCR (qRT-PCR) assays were performed using the 7300 Realtime PCR system (Applied Biosystems) as described previously by Bieche *et al.* [16] using the SYBR Green Master Mix (Applied Biosystems) as the amplification detector and the rpoA gene as the endogenous control. The absence of DNA in the samples was confirmed by classical PCR with primers CPYFLA_1 5′-GGATTTCGTATTAACACAAATGGTGC-3′ and CPYFLA_2 5′ CTGTAGTAATCTTAAAACATTTTG-3′ amplifying 1700 bp of the gene flaA. Gene-specific primers used for qRT-PCR were designed according to the corresponding gene sequences of the identified proteins (Table 3). Three independent RNA extractions with four replicates for each gene were performed for each condition.

**Table 3:** Primers used in this study for gene expression quantification using qRT-PCR.

Primer name	Sequence 5′-3′	Amplicon length (bp) (ref)
**rpoAQ-fw**	CGAGCTTGCTTTGATGAGTG	109 (Garénaux *et al.*)
**rpoAQ-rev**	AGTTCCCACAGGAAAACCTA	
**cadF-fw**	TGCTGATACTCGTGCAACTC	112 (Garénaux *et al.*)
**cadF-rev**	ACCAAAATGACCTTCCAAAG	
**cjj0854-fw**	GGTAGCGTTTTAAGCGTGGA	106
**cjj0854-rev**	TTTTTACAGCTTGGGTAATTTCTTTT	
**cjj0275-fw**	TCATGCTGCTCGTGAAGAAG	106
**cjj0275-rev**	TGCAGCTTTTGCGTTAAATG	
**dnaj1-fw**	TATGTTCCCCGCCTTTAACA	109
**dnaj1-rev**	CCGCGGTTTTTAAATTCTTG	
**cjj0093-fw**	TAGCCTTTGCCAAACCTGAT	116
**cjj0093-rev**	TATACCGCACATTCCACCAA	
**peb4-fw**	ACAGATGCTGCTTTCGCACT	108
**peb4-rev**	TTGACCTTTAGCCTGCGAAT	

fw: forward, rev: reverse, bp: base pair.

### Statistical analyses

The results from adhesion, motility and qRT-PCR, including assays and conditions, were analyzed using Statgraphics Plus 5.1 software (StatPoint Inc., Herndon, Virginia, USA). With the confirmation of a normal distribution for each data set, significant differences were determined using two-sided Student's t-test comparisons at a 5% significance level.

## Supporting Information

Figure S1 Principal Component Analysis performed on the complete data set of the 14 2-DE gels for IMPs-enriched fraction (A) and 13 2-DE gels for OMPs-enriched fraction (B). Blue circles correspond to proteins of oxygen-acclimated cells and pink circles to proteins of microaerobically grown cells (control).
(DOC)

Figure S2 **Alignment of CadF protein sequences from different strains of** ***C. jejuni*** **using software ClutalX2.** Frames highlight the suspected adhesion to fibronectin site **(FRLS)** and the two potential protease sites **(SL)** and **(GF)**.
(DOC)

Figure S3 **Protein coverage and matched peptides from the three protein forms of CadF (CadF-1, CadF-2 and CadF-3) which abundance was modulated under oxygen-acclimation conditions.** Matched peptides are indicated in red.
(DOC)
