# Supplementary figures and images for: Enhanced Adhesion of Campylobacter jejuni to Abiotic Surfaces Is Mediated by Membrane Proteins in Oxygen-Enriched Conditions
Source: PLoS One. 2012 Sep 28;7(9):e46402. doi: 10.1371/journal.pone.0046402 (PMC3460892; doi:10.1371/journal.pone.0046402)

*Sulaeman et al.*

Supplementary Figure S1


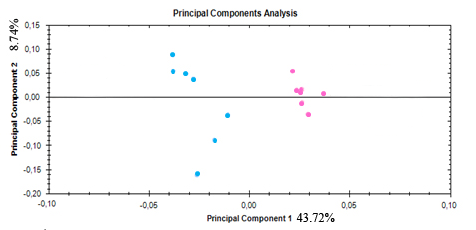


**A (IMP-enriched fraction)**


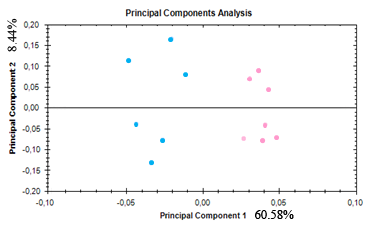


**B (OMP-enriched fraction)**

Supplement: Figure S1 — Principal Component Analysis performed on the complete data set of the 14 2-DE gels for IMPs-enriched fraction (A) and 13 2-DE gels for OMPs-enriched fraction (B). Blue circles correspond to proteins of oxygen-acclimated cells and pink circles to proteins of microaerobically grown cells (control). (DOC) [file pone.0046402.s001.doc]

*Sulaeman et al.*

Supplementary Figure S2


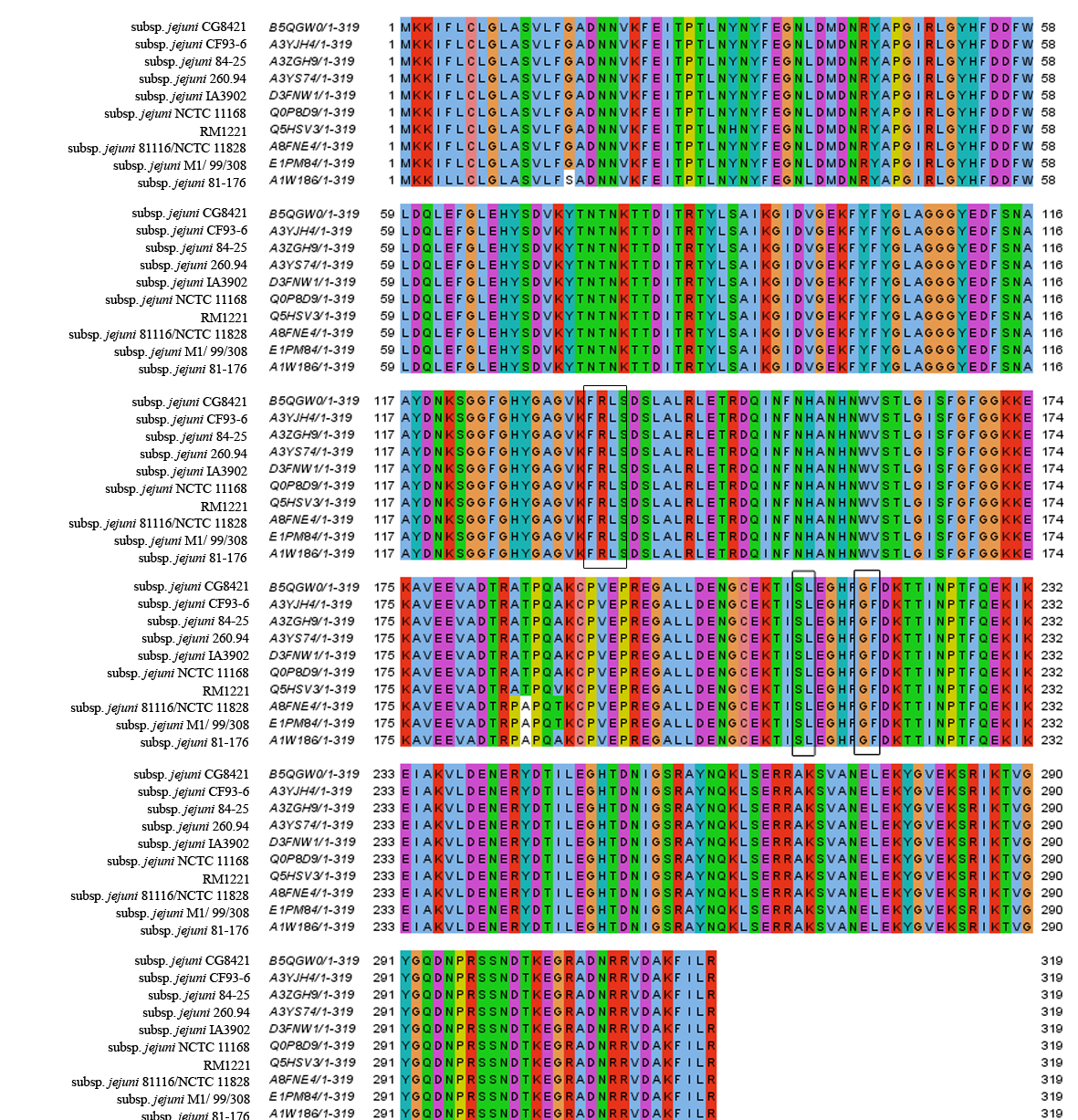

Supplement: Figure S2 — Alignment of CadF protein sequences from different strains of C. jejuni using software ClutalX2. Frames highlight the suspected adhesion to fibronectin site (FRLS) and the two potential protease sites (SL) and (GF). (DOC) [file pone.0046402.s002.doc]
