# Supplementary material for: Enhanced Adhesion of Campylobacter jejuni to Abiotic Surfaces Is Mediated by Membrane Proteins in Oxygen-Enriched Conditions
Source: PLoS One. 2012 Sep 28;7(9):e46402. doi: 10.1371/journal.pone.0046402 (PMC3460892; doi:10.1371/journal.pone.0046402)
Supplement: Figure S3 — Protein coverage and matched peptides from the three protein forms of CadF (CadF-1, CadF-2 and CadF-3) which abundance was modulated under oxygen-acclimation conditions. Matched peptides are indicated in red. (DOC) [file pone.0046402.s003.doc]

*Sulaeman et al.*

Supplementary Figure S3

**CadF-1**

Match to: **gi|121612147** Score: **202**

**fibronectin-binding protein [Campylobacter jejuni subsp. jejuni 81-176]**

[gi|87249779](http://www.ncbi.nlm.nih.gov/entrez/query.fcgi?cmd=search&db=protein&doptcmdl=genpept&tool=mascot&term=gi|87249779%5Baccn%5D) from [Campylobacter jejuni subsp. jejuni 81-176](http://www.ncbi.nlm.nih.gov/Taxonomy/Browser/wwwtax.cgi?lvl=0&id=354242)

Sequence Coverage: **12% (6 matched peptides)**

Nominal mass (M_r_): **35967**; Calculated pI value: **5.89**

**1** MKKILLCLGL ASVLFSADNN VKFEITPTLN YNYFEGNLDM DNRYAPGIRL

**51** GYHFDDFWLD QLEFGLEHYS DVKYTNTNKT TDITRTYLSA IKGIDVGEKF

**101** YFYGLAGGGY EDFSNAAYDN K**SGGFGHYGA GVK**FRLSDSL ALRLETRDQI

**151** NFNHANHNWV STLGISFGFG GKK**EKAVEEV ADTRPAPQAK** CPVEPR**EGAL**

**201 LDENGCEK**TI SLEGHFGFDK TTINPTFQEK IKEIAKVLDE NERYDTILEG

**251** HTDNIGSRAY NQKLSERRAK SVANELEKYG VEKSRIKTVG YGQDNPRSSN

**301** DTKEGRADNR RVDAKFILR

**CadF-2**

Match to: **gi|121612147** Score: **540**

**fibronectin-binding protein [Campylobacter jejuni subsp. jejuni 81-176]**

[gi|87249779](http://www.ncbi.nlm.nih.gov/entrez/query.fcgi?cmd=search&db=protein&doptcmdl=genpept&tool=mascot&term=gi|87249779%5Baccn%5D) from [Campylobacter jejuni subsp. jejuni 81-176](http://www.ncbi.nlm.nih.gov/Taxonomy/Browser/wwwtax.cgi?lvl=0&id=354242)

Sequence Coverage: **34% (13 matched peptides)**

Nominal mass (M_r_): **35967**; Calculated pI value: **5.89**

**1** MKKILLCLGL ASVLFSADNN VKFEITPTLN YNYFEGNLDM DNRYAPGIRL

**51** GYHFDDFWLD QLEFGLEHYS DVKYTNTNKT TDITR**TYLSA IK**GIDVGEKF

**101** YFYGLAGGGY EDFSNAAYDN K**SGGFGHYGA GVK**FR**LSDSL ALR**LETRDQI

**151** NFNHANHNWV STLGISFGFG GKK**EKAVEEV ADTRPAPQAK** CPVEPREGAL

**201** LDENGCEK**TI SLEGHFGFDK TTINPTFQEK** IKEIAK**VLDE NERYDTILEG**

**251 HTDNIGSR**AY NQKLSERRAK **SVANELEKYG VEK**SRIK**TVG YGQDNPR**SSN

**301** DTKEGRADNR RVDAKFILR

**CadF-3**

Match to: **gi|121612147** Score: **235**

**fibronectin-binding protein [Campylobacter jejuni subsp. jejuni 81-176]**

[gi|87249779](http://www.ncbi.nlm.nih.gov/entrez/query.fcgi?cmd=search&db=protein&doptcmdl=genpept&tool=mascot&term=gi|87249779%5Baccn%5D) from [Campylobacter jejuni subsp. jejuni 81-176](http://www.ncbi.nlm.nih.gov/Taxonomy/Browser/wwwtax.cgi?lvl=0&id=354242)

Sequence Coverage: **14% (6 matched peptides)**

Nominal mass (M_r_): **35967**; Calculated pI value: **5.89**

**1** MKKILLCLGL ASVLFSADNN VKFEITPTLN YNYFEGNLDM DNRYAPGIRL

**51** GYHFDDFWLD QLEFGLEHYS DVKYTNTNKT TDITRTYLSA IKGIDVGEKF

**101** YFYGLAGGGY EDFSNAAYDN K**SGGFGHYGA GVK**FR**LSDSL ALR**LETRDQI

**151** NFNHANHNWV STLGISFGFG GKKEK**AVEEV ADTRPAPQAK** CPVEPR**EGAL**

**201 LDENGCEK**TI SLEGHFGFDK TTINPTFQEK IKEIAKVLDE NERYDTILEG

**251** HTDNIGSRAY NQKLSERRAK SVANELEKYG VEKSRIKTVG YGQDNPRSSN

**301** DTKEGRADNR RVDAKFILR
